# Prediction and curation of missing biomedical identifier mappings with Biomappings

**DOI:** 10.1093/bioinformatics/btad130

**Published:** 2023-03-14

**Authors:** Charles Tapley Hoyt, Amelia L Hoyt, Benjamin M Gyori

**Affiliations:** Laboratory of Systems Pharmacology, Harvard Medical School, Boston, MA 02115, United States; Beth Israel Deaconess Medical Center, Boston, MA 02215, United States; Laboratory of Systems Pharmacology, Harvard Medical School, Boston, MA 02115, United States

## Abstract

**Motivation:**

Biomedical identifier resources (such as ontologies, taxonomies, and controlled vocabularies) commonly overlap in scope and contain equivalent entries under different identifiers. Maintaining mappings between these entries is crucial for interoperability and the integration of data and knowledge. However, there are substantial gaps in available mappings motivating their semi-automated curation.

**Results:**

Biomappings implements a curation workflow for missing mappings which combines automated prediction with human-in-the-loop curation. It supports multiple prediction approaches and provides a web-based user interface for reviewing predicted mappings for correctness, combined with automated consistency checking. Predicted and curated mappings are made available in public, version-controlled resource files on GitHub. Biomappings currently makes available 9274 curated mappings and 40 691 predicted ones, providing previously missing mappings between widely used identifier resources covering small molecules, cell lines, diseases, and other concepts. We demonstrate the value of Biomappings on case studies involving predicting and curating missing mappings among cancer cell lines as well as small molecules tested in clinical trials. We also present how previously missing mappings curated using Biomappings were contributed back to multiple widely used community ontologies.

**Availability and implementation:**

The data and code are available under the CC0 and MIT licenses at https://github.com/biopragmatics/biomappings.

## 1 Introduction

Standardizing the identification of small molecules, proteins, and other biomedical entities is an important step in creating and maintaining findable, accessible, interoperable, and reusable (FAIR) (Wilkinson et al. 2016) data in the life sciences. Resources that catalog and provide identifiers for such entities are essential for this effort. Such “identifier resources” include ontologies, taxonomies, and other controlled vocabularies, e.g. the Human Disease Ontology (DO) (Schriml et al. 2021), Medical Subject Headings (MeSH) (Rogers 1963), and the Chemical Entities of Biological Interest (ChEBI) (Hastings et al. 2016). However, many identifier resources overlap in scope and include equivalent entities with different identifiers. For example, the small molecule cyclin-dependent kinase inhibitor “alsterpaullone” appears in several chemical- and drug-related identifier resources including ChEBI as chebi:138488 and MeSH as mesh:C120793. Merging equivalent entities is crucial for tasks dependent on data integration like ontology merging (Lambrix and Tan 2008; Geleta et al. 2022; Guo et al. 2022), entity linking (Gyori et al. 2022), construction of knowledge graphs (Himmelstein et al. 2017; Nicholson and Greene 2020; Friedrichs 2021), and automated systems biology model assembly (Gyori et al. 2017; Bachman et al. 2023). More generally, mapping between equivalent identifiers from different identifier resources is a ubiquitous task across computational life science analyses, workflows, and tools.

A “mapping” (often referred to as “ontology mapping” or “semantic mapping”) represents a relationship between two entities in different identifier resources using a specific predicate such as one representing an exact match (i.e. when the entities can be used interchangeably), a broad match (i.e. when one entity is a super-class of the other), or a narrow match (i.e. when one entity is a subclass of the other). Each mapping can also carry additional metadata representing provenance for its creation. We refer to Figure 2 of Matentzoglu et al. (2022a) for a more detailed description of mappings. High-quality, semantically rich equivalence mappings are required to support merging and converting between identifiers from different resources. Therefore, many identifier resources provide equivalence mappings to one or more other resources. We refer to mappings provided directly by an identifier resource as “primary mappings.” Such mappings are typically curated by the maintainers of the resource and are provided along with entries in the resource in the form of cross-references. For example, the HUGO Gene Nomenclature Committee (HGNC) (Yates et al. 2017) provides mappings from its identifiers for human genes to the Entrez gene database (Maglott et al. 2011), Ensembl (Zerbino et al. 2018), and several other identifier resources. Similarly, ontologies like the Mondo Disease Ontology (MONDO) (Vasilevsky et al. 2022) curate and distribute mappings to other identifier resources including DO and MeSH.

Though primary mappings from identifier resources are often available, a survey from Laadhar et al. (2020) of mappings in life science ontologies highlights several widespread issues such as the use of unspecific predicates (e.g. oboInOwl:hasDbXRef), the lack of standardization of the syntax and semantics of the targets of mappings, and a lack of detailed provenance metadata. The Simple Standard for Sharing Ontological Mappings (SSSOM) (Matentzoglu et al. 2022a) was recently developed to provide a standardized format for mappings and thereby increase their reusability and interoperability. While SSSOM supports disseminating mappings through a common standard, it does not in itself provide a solution for identifying and curating missing mappings.

Several services aggregate, process, and redistribute mappings including BridgeDB (van Iersel et al. 2010), TogoID (Ikeda et al. 2022), and the Ontology Mapping Service (https://github.com/EBISPOT/OXO). However, these services only draw from primary mappings provided by identifier resources and are therefore unable to address gaps, lack of specificity, and lack of rich provenance metadata in these resources.

Despite existing curation and aggregation efforts, there still exist gaps in mappings between major resources (e.g. ChEBI and MeSH both provide identifiers for small molecules but neither provides mappings to the other) and mappings that are provided are often incomplete (e.g. there are many gaps in mapping disease terms between MONDO and MeSH). Several automated methodologies have been proposed to predict missing mappings, including algorithms exploiting lexical similarity (Ghazvinian et al. 2009), ones based on logical/structural alignment of resources (Jiménez-Ruiz and Cuenca Grau 2011), and others based on machine learning (Berrendorf et al. 2020). However, beyond routine benchmarking, mappings produced by such automated mapping algorithms are not systematically reviewed for correctness and then contributed back to the primary sources to which the mappings apply, ultimately resulting in limited impact on the state of existing resources. Further, existing automated mapping approaches often do not provide interfaces for curating (e.g. reviewing, confirming, and rejecting) predicted mappings, storing important metadata (e.g. mapping confidence), and maintaining curation artifacts in accessible ways (e.g. via public version control). For example, the Ontology Alignment Evaluation Initiative (https://oaei.ontologymatching.org) has used the same task of aligning the Foundational Model of Anatomy Ontology (Rosse and Mejino 2003) and SNOMED-CT (Donnelly et al. 2006) for more than 15 years [see Bodenreider and Zhang (2006) and Wang and Hu (2022)]. However, the manuscripts published on the task only focus on method development and neither predict novel mappings, curate them, nor contribute them back to the upstream identifier resources. Overall, existing work does not provide a workflow for finding, predicting, and curating missing mappings.

To address this, we introduce Biomappings, a framework for semi-automatically creating and maintaining mappings in a public, version-controlled repository. Biomappings combines multiple contributions: (i) a “curation cycle” workflow for creating mappings; (ii) an extensible pipeline for automatically predicting missing mappings between resources, and automatically detecting inconsistencies; (iii) a web interface for reviewing and curating predicted mappings; and (iv) a public, version-controlled repository of predicted and curated mappings.

Biomappings currently makes available ∼9.2 thousand reviewed mappings and ∼40 thousand predicted ones. Mappings are associated with necessary metadata in an intuitive tab-delimited format, licensed permissively to encourage community contributions and restriction-free integration back into primary identifier resources. In addition to novel mappings, Biomappings provides an extensible lexical mapping prediction pipeline based on Gilda (Gyori et al. 2022), and a web-based interface for curation of predictions and adding manually constructed mappings. The mappings themselves, functions supporting the programmatic creation and usage of mappings, the web-based curation interface, as well as several workflow examples for generating new mappings are made available in the open-source “biomappings” Python package.

We demonstrate the utility of Biomappings in three case studies. First, we used Biomappings to predict and curate mappings for cell lines in the Cancer Cell Line Encyclopedia (CCLE) (Ghandi et al. 2019) to two other resources providing identifiers for cell lines. Biomappings added a total of 684 novel mappings that could not be inferred from existing mappings (more than a 70% increase). These mappings crucially improve the interoperability between databases describing the characteristics and measurements of cancer cell lines.

Second, we used Biomappings to predict and curate mappings between MeSH and ChEBI, both of which contain entries for chemicals of biological interest but lack any mappings to each others’ entries. We show that the 2909 mappings added by Biomappings enable mapping chemicals (listed using MeSH identifiers) in 100 342 ClinicalTrials.gov trials to ChEBI identifiers, thereby enabling the integration of clinical trials data with other ChEBI-aligned resources.

Third, we used Biomappings to predict and curate missing mappings between four widely used Open Biological and Biomedical Ontologies (OBO) (Jackson et al. 2021) and MeSH. We then contributed 1378 confirmed mappings back to the primary OBO resources through GitHub pull requests. These contributions have the potential for high impact given the ubiquity of these ontologies in various workflows, tools, and analyses.

Biomappings is available through a web portal at https://biopragmatics.github.io/biomappings and as a Python software package at https://pypi.org/project/biomappings. All underlying data, code, and governance documentation are accessible through GitHub at https://github.com/biopragmatics/biomappings under the MIT and CC0 licenses and are archived on Zenodo (https://doi.org/10.5281/zenodo.7307938).

## 2 Materials and methods

### 2.1 The Biomappings curation cycle

**Figure 1.**
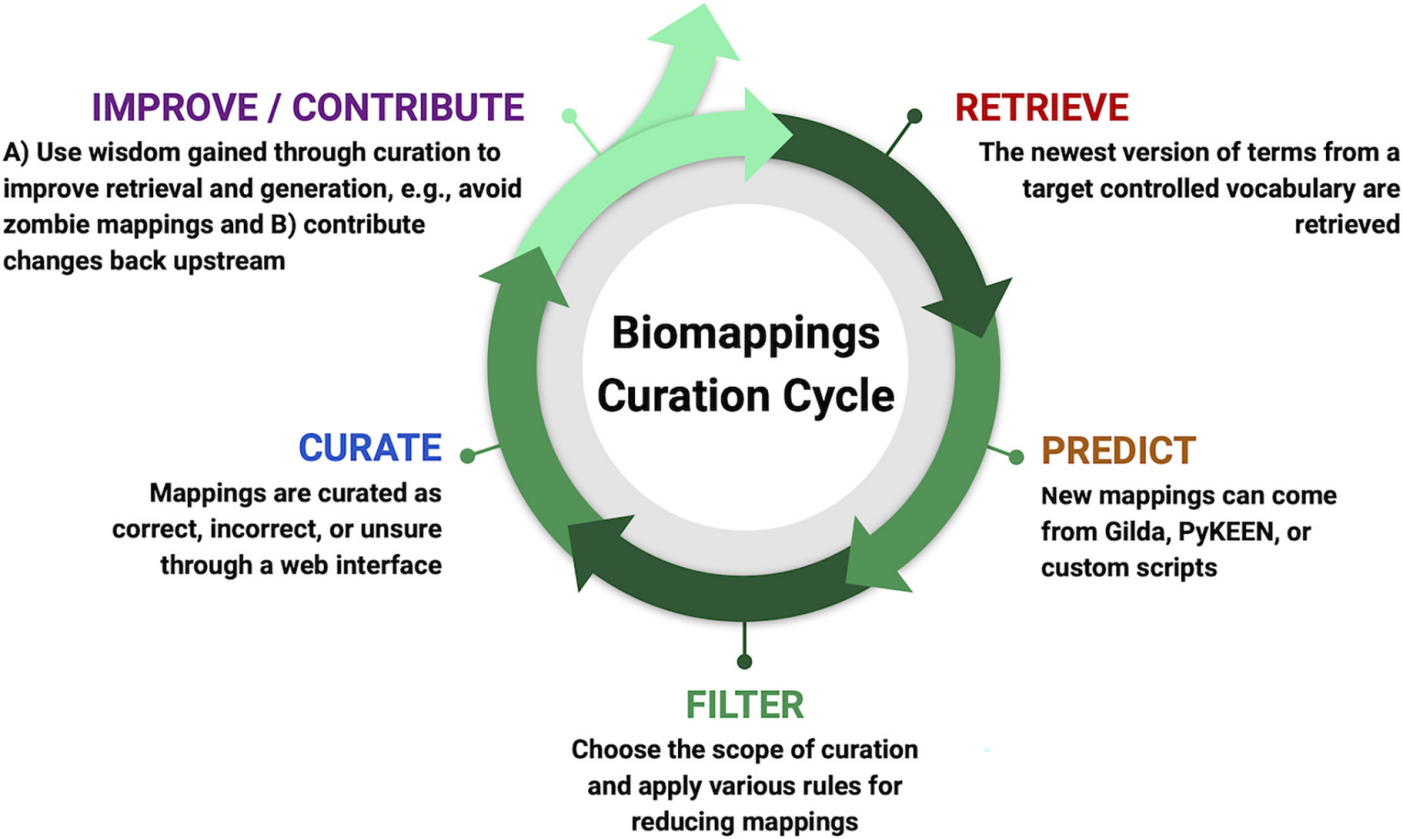
The Biomappings curation cycle supports sustainable, efficient curation of high-quality mappings between identifier resources, consisting of five steps.

We propose a novel, semi-automated approach to curation and maintenance of mappings supported by Biomappings (Fig. 1).

The first step of the cycle comprises *retrieval* and preprocessing of target identifier resources, including any existing mappings between the resources. We automate this process for ontologies by using the Bioregistry (Hoyt et al. 2022) to locate the ontology (i.e. with a URL) and ROBOT (Jackson et al. 2019) to parse it. Similarly, we use custom automated preprocessing workflows in PyOBO (https://github.com/pyobo/pyobo) for other identifier resource types (e.g. databases like HGNC).

The second step comprises *generating predictions* through lexical similarity or other approaches (see Section 2.2). This step is often mediated by task-specific scripts which focus on generating mappings between two target identifier resources. At this stage, predictions for mappings that have already been manually curated in Biomappings or ones made available by other sources can be excluded to ensure predictions are novel.

The third step comprises choosing a scope for manual review, i.e. *filtering* predictions to a desired subset and applying additional filters. For instance, one may filter for predicted mappings between two specific identifier resources to focus curation on.

The fourth step is to review and *curate* predicted mappings as positive (i.e. true), negative (i.e. false), or unsure. In Biomappings, this step is mediated by a web interface described in Section 2.3.

The fifth step involves *improving* the first four steps of the cycle based on insights gained from curation, as well as *contributing* curated mappings to external resources. For example, manual inspection of predictions may reveal systematic errors in prediction that can be used to improve preprocessing or update the filtering step in order to reduce curation burden. Concurrently, the novel mappings can be contributed to external resources to increase their visibility and impact (we demonstrate this in Section 4.3).

This curation workflow may support one of several goals such as (1) the exhaustive curation of mappings between identifiers in two identifier resources, (2) a task-oriented curation goal to maximize return on fixed curation effort, often prioritized or driven by an external task-specific need, (3) analysis or quality assurance-driven curation such as the example described in Section 2.4.2, or (4) open-ended/interest-driven curation.

### 2.2 Generating predictions

We employ a lexical matching workflow in the case studies presented in Section 4. This relies on labels and synonyms associated with entities for predicting mappings. The workflow is implemented using Gilda (Gyori et al. 2022), a fast and extensible named entity normalization system. Gilda takes an unnormalized string representing a biomedical entity as input (e.g. “k-ras”) and returns a list of ranked, scored matching ontology terms (e.g. HGNC:6407 representing the KRAS gene). Initial retrieval of possible matches is done based on string normalization (e.g. “Amyloid-β” becomes “amyloid-beta”) after which possible matches are scored and ranked. Scoring is based on an extension of Allen et al. (2015) and takes into account string variations between the unnormalized strings including dashes, capitalization patterns, Greek letters, and their spelled out forms. When used to predict mappings between two identifier resources, Gilda first generates an index of the labels and synonyms for entities in one of the two identifier resources. Second, Gilda takes labels and synonyms in the second identifier resource and finds ranked/scored matches to the first one.

Lexical matching has the advantage of being computationally inexpensive, highly explainable, and ultimately easy to curate. However, we note that the Biomappings workflow is able to support other methodologies such as knowledge graph-based matching workflows (see Supplement), structural matching (e.g. that exploits ontology hierarchy, see Supplement), chemical structure-based matching, or other custom workflows. Following generation, predicted mappings are filtered to remove predictions that appear in primary identifier resources or have already been curated in Biomappings in order to reduce duplicate curation. Importantly, Biomappings also captures provenance about how mappings are generated irrespective of the methodology.

### 2.3 Web interface for curating mappings

**Figure 2.**
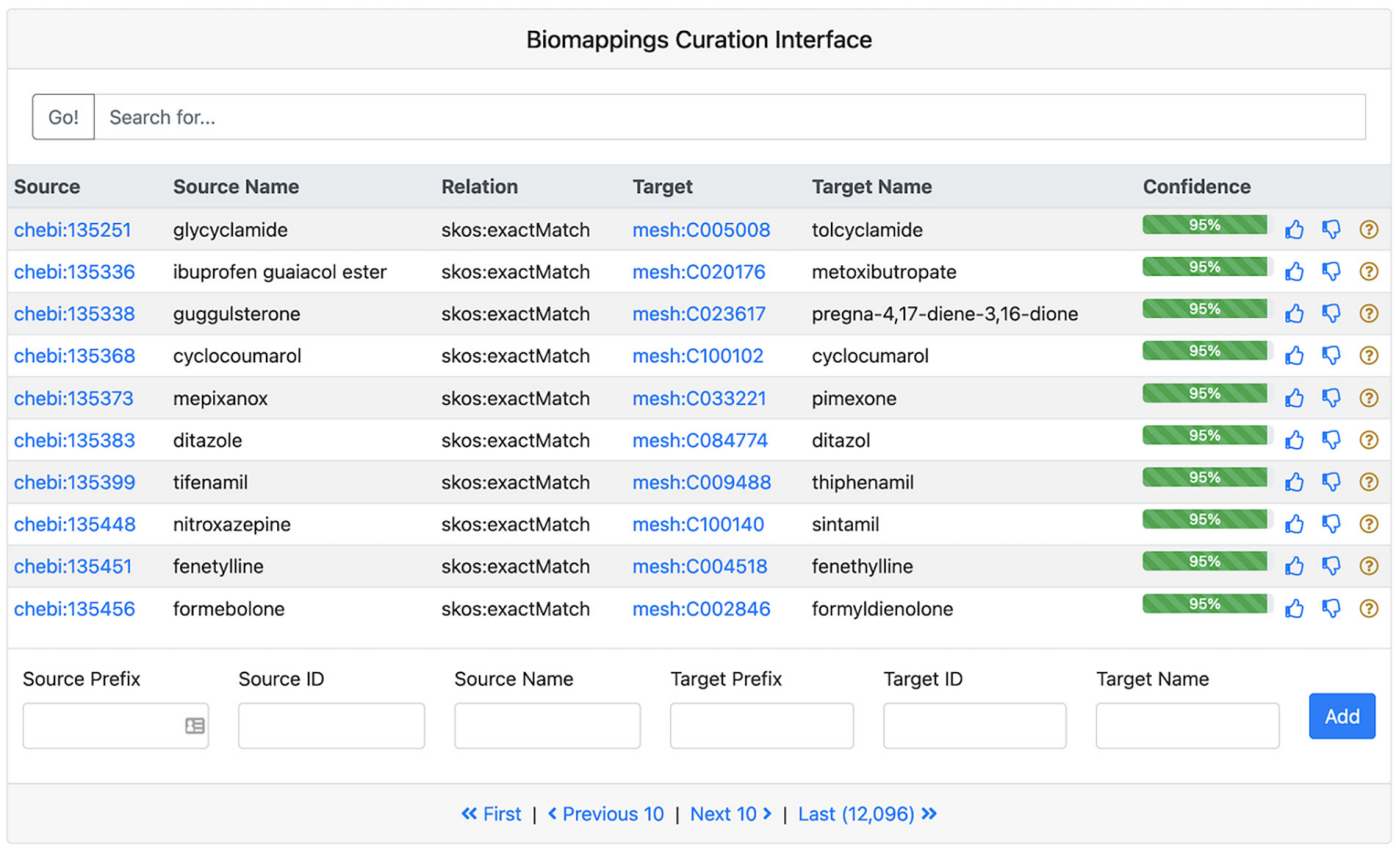
A screenshot of the Biomappings curation interface filtered to ChEBI and MeSH mappings showcases mappings of varying degrees of difficulty to curate. For example, some are exact matches, some are close matches, and some are matched due to synonyms.

Biomappings provides a locally deployable web-based interface for browsing, reviewing, and curating predicted mappings (Fig. 2). It displays a paginated view of the subject, predicate, object, and confidence of each predicted mapping that can be searched by the compact uniform resource identifier (CURIE) for an entity (i.e. of the form *<prefix>*:*<identifier>*), by entity name, and by resource to support restricted curation scopes. On the right-hand side, it displays buttons for curating each prediction as positive, negative, or unsure. After each curation, the interface updates the relevant resource files and includes provenance about the curation such as the curator’s Open Researcher and Contributor Identifier (ORCID). It then communicates with git to create the appropriate commits that can be pushed to GitHub. Finally, the bottom of the interface also includes a bar for inputting novel curations not included in the predictions.

### 2.4 Quality assurance

Biomappings uses a combination of social and technical workflows to maintain high data quality and integrity.

#### 2.4.1 Version control and continuous testing

Biomappings uses git for version control to track all changes and mediate releases via Zenodo. Second, it uses GitHub as a technical platform to host and distribute the project’s code and data openly and as a social platform to enable discussion and external contribution through pull requests. Third, it uses GitHub Actions as a continuous integration and continuous delivery system to apply data quality checks [e.g. all mappings’ prefixes and local unique identifiers are compliant with the Bioregistry (Hoyt et al. 2022)]. Further, several summaries (e.g. charts, tables, and website), analyses (see Section 2.4.2), and artifacts (see Section 3.1) are automatically regenerated when a pull request is merged and archived on Zenodo (https://doi.org/10.5281/zenodo.7307938).

#### 2.4.2 Automated consistency checking of mappings

**Figure 3.**
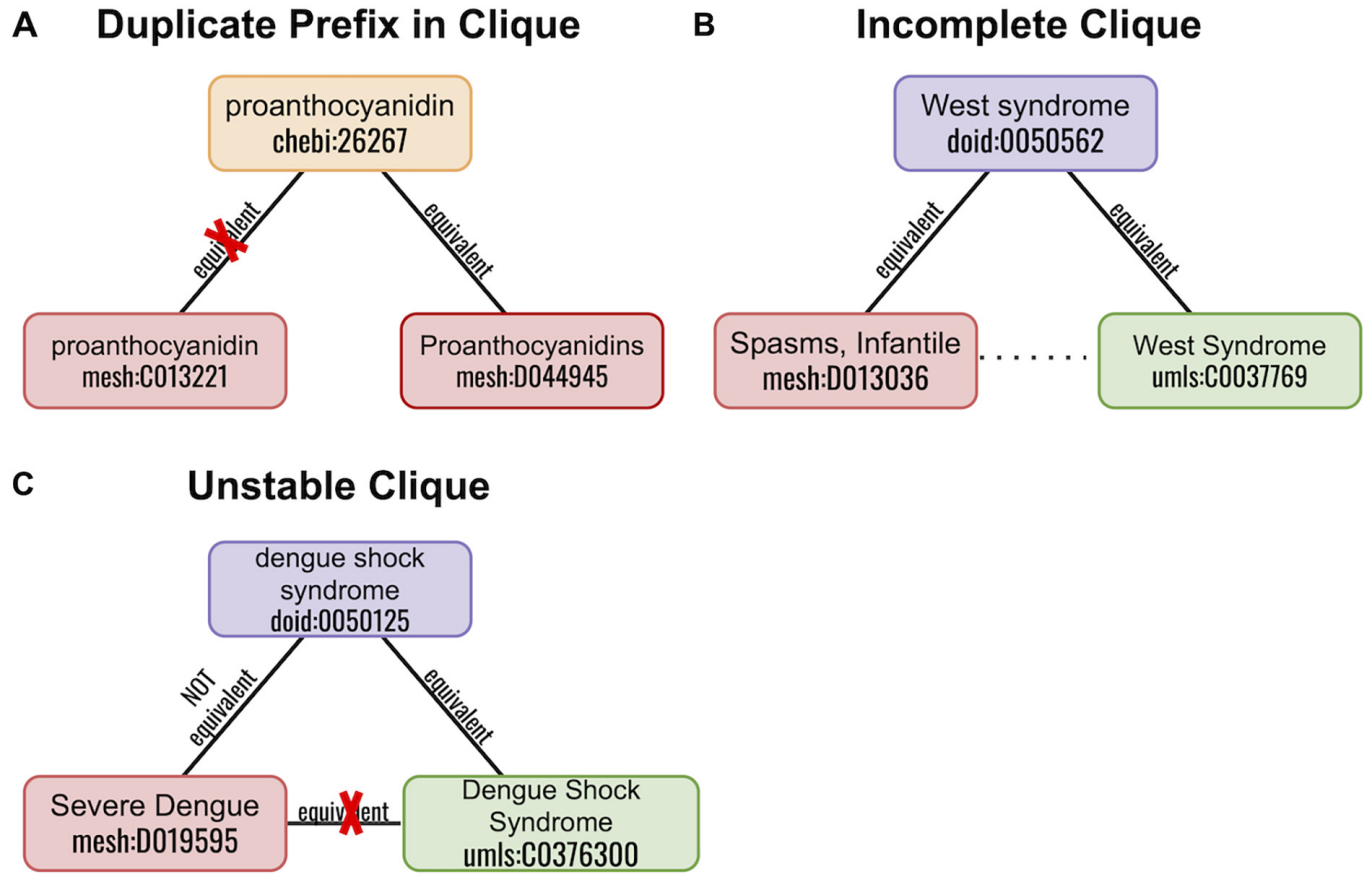
Three motifs identified by graph-theoretic methods for quality assurance. The red X represents a mapping (whether positive or negative) that was incorrectly curated and should be removed. (**A**) A prefix appearing twice in a triangle of positive mappings signifies that one node and all of its incident edges is incorrectly mapped. (**B**) A missing edge in a path of positive mappings with three nodes suggests the existence of a high confidence mapping. (**C**) A triangle with a negative mapping and two positive mappings implies one of the positive mappings is incorrect.

Biomappings implements three graph-theoretic approaches for identifying incorrectly curated, inconsistent, or missing mappings. This serves as an automated quality check to maintain the global consistency of the resource. First, a labeled, undirected graph is constructed from the union of positive and negative exact mappings. Then, certain predefined motifs are found as shown in Fig. 3 to detect inconsistencies.

The “duplicate prefix in clique” motif contains a set of nodes connected by equivalence relationships (e.g. skos:exactMatch) where two or more nodes originate from the same identifier resource (Fig. 3A). In the example, two entities from MeSH [i.e. “proanthocyanidin” (mesh:C013221) and “Proanthocyanidins” (mesh:D044945)] appear in the same clique. Upon further inspection, “Proanthocyanidins” is found to represent a class of chemicals with similar structures while “proanthocyanidin” represents a specific, prototypical instance of the class. Because the other entities in the clique refer to the class of chemicals, the equivalence relations between “proanthocyanidin” (i.e. the specific instance) and the other entities in the clique should be removed. This removal, however, does not preclude the existence of other relationships, such as parent/child relationships. Such motifs can also help identify more generic properties of the identifier resources that lead to curation errors, such as the pluralization schemes of its chemical classes.

The “incomplete clique” motif contains a set of nodes that are connected by equivalence relationships, but some nodes are not connected (Fig. 3B). This motif provides support for curating additional equivalence mappings via the transitivity of equivalence. In the example, a mapping between “Spasms, Infantile” (mesh:D013036), and “West Syndrome” (umls:C0037769) could be curated.

The “unstable clique” motif contains a set of nodes that are connected by equivalence relationships, but two of the nodes are also curated with a negative mapping (Fig. 3). This motif suggests that one (or more) of the equivalence or negative mappings are incorrect and should be removed. In the example, the equivalence between “Severe Dengue” (mesh:D019595) and “Dengue Shock Syndrome” (umls:C0376300) is incorrect. These analyses are run on all changes to the Biomappings database using GitHub Actions as a continuous integration service whose results are posted to https://biopragmatics.github.io/biomappings.

## 3 Results

Biomappings (v0.3.0) contains 9274 positive mappings, 1215 negative mappings, 67 unsure mappings, and 40 691 predicted mappings of 6 types across 27 identifier resources curated by 6 individuals (Table 1, see also detailed summary in Supplementary Fig. S2). These mappings were generated through a combination of prioritized curation (e.g. motivated by creating mappings to support a specific downstream task), open-ended curation (e.g. motivated by interest in a specific prefix or search term or motivated by curating the highest confidence mappings), and exhaustive curation (e.g. motivated by completing alignments between two or more identifier resources). Examples of each can be found in Section 4.

**Table 1.** Biomappings resource files.

Curated	Description	Count
Yes	Human-curated positive (i.e. true) mappings	9274
Yes	Human-curated “non-trivial” negative (i.e. false) mappings	1215
Yes	Mappings that have been checked but not yet decided	67
No	Automatically predicted mappings	40 691

*Note*: Each row corresponds to a distinct TSV file in version control on GitHub.

While Biomappings is generally able to use any predicate encoded as a CURIE, mappings typically use the Simple Knowledge Organization System (SKOS) vocabulary to denote matches in the sense of information retrieval. The three most common predicates that are useful for curating mappings are skos:exactMatch for terms that can be used interchangeably, skos:broadMatch for when the object term is a super-class of the subject, and skos:narrowMatch for when the object term is a subclass of the subject. Three additional relations appear in Biomappings (v0.3.0) for species differentia [e.g. for mapping species-generic and species-specific pathways in KEGG (Kanehisa et al. 2017)], for homologs [e.g. for mapping related species-specific pathways in WikiPathways (Martens et al. 2021)], and for connecting different ionization states (e.g. conjugate acid and conjugate base) of small molecules.

### 3.1 Availability

Biomappings stores mappings in four tab-separated values (TSV) files corresponding to the positive (i.e. true), (non-trivially) negative (i.e. false), unsure, and predicted mappings (Table 1) that can be manually edited directly, modified via web-based manual curation, or extended with new predictions programmatically. Each stores rich metadata about the source, target, mapping type, as well as its associated provenance. Further, the predicted mappings include confidence assessments and additional provenance about how they were generated. These are collated into a single SSSOM document which fully represents all provenance, can be used to generate additional exports (e.g. JSON and RDF), and can be readily consumed by curation workflows such as those in the Ontology Development Kit (Matentzoglu et al. 2022b) for developing ontologies. These artifacts are re-generated by the continuous integration workflow triggered on all data changes in the repository. Further, these mappings can be accessed via the biomappings Python package, installable through the Python Package Index (PyPI). Finally, the mappings are available as an interactive network on the Network Data Exchange (NDEx) (Pratt et al. 2015) under https://bioregistry.io/ndex:402d1fd6-49d6-11eb-9e72-0ac135e8bacf.

### 3.2 Governance and sustainability

Biomappings is built using open source code and open data distributed under a permissive license (MIT and CC0) in a public, version-controlled repository that is archived on Zenodo in order to encourage community reuse and incorporation into the upstream resources that it describes. It leverages public infrastructure and automation to support its maintenance and extension. It has well-defined contribution guidelines (https://biopragmatics.github.io/biomappings/contributing) and a governance model (https://biopragmatics.github.io/biomappings/governance) that enable contributions directly from the broader community to support the project’s longevity. Finally, Biomappings has a transparent attribution model that associates all mappings with the ORCID identifier of the curator. These are summarized on the auto-generated summary website and also on APICURON (Hatos et al. 2021).

## 4 Case studies

We present three case studies demonstrating the utility of Biomappings. In Section 4.1, we describe generating and curating exhaustive mappings between several identifier resources for cancer cell lines. In Section 4.2, we describe taking a prioritized approach toward generating and curating mappings between ChEBI and MeSH in order to support data integration with clinical trials data. Finally, in Section 4.3, we describe predicting and curating missing MeSH mappings for several OBO ontologies, then contribute the results back to these ontologies.

### 4.1 Mapping missing cancer cell line identifiers

Several identifier resources have been constructed to describe cell lines in order to support the generation of databases describing their characteristics and associated experimental measurements. For example, the CCLE contains a detailed genetic and pharmacological characterization of hundreds of cancer cell lines constituting models of various human cancers. Such detailed, large-scale databases have proven useful in the prediction of anticancer drug sensitivity (Barretina et al. 2012) and preclinical testing (Wilding and Bodmer 2014).

We were interested in integrating experimental data from CCLE in a dialog system, but because CCLE provides an identifier resource that does not contain lexicalizations (i.e. names and synonyms), it was necessary to map CCLE to external identifier resources in order to use their respective lexicalizations. Cellosaurus (Bairoch 2018) and the Experimental Factor Ontology (EFO) (Malone et al. 2010) were chosen for this purpose because of their high-quality curation and detailed lexicalizations.

**Figure 4.**
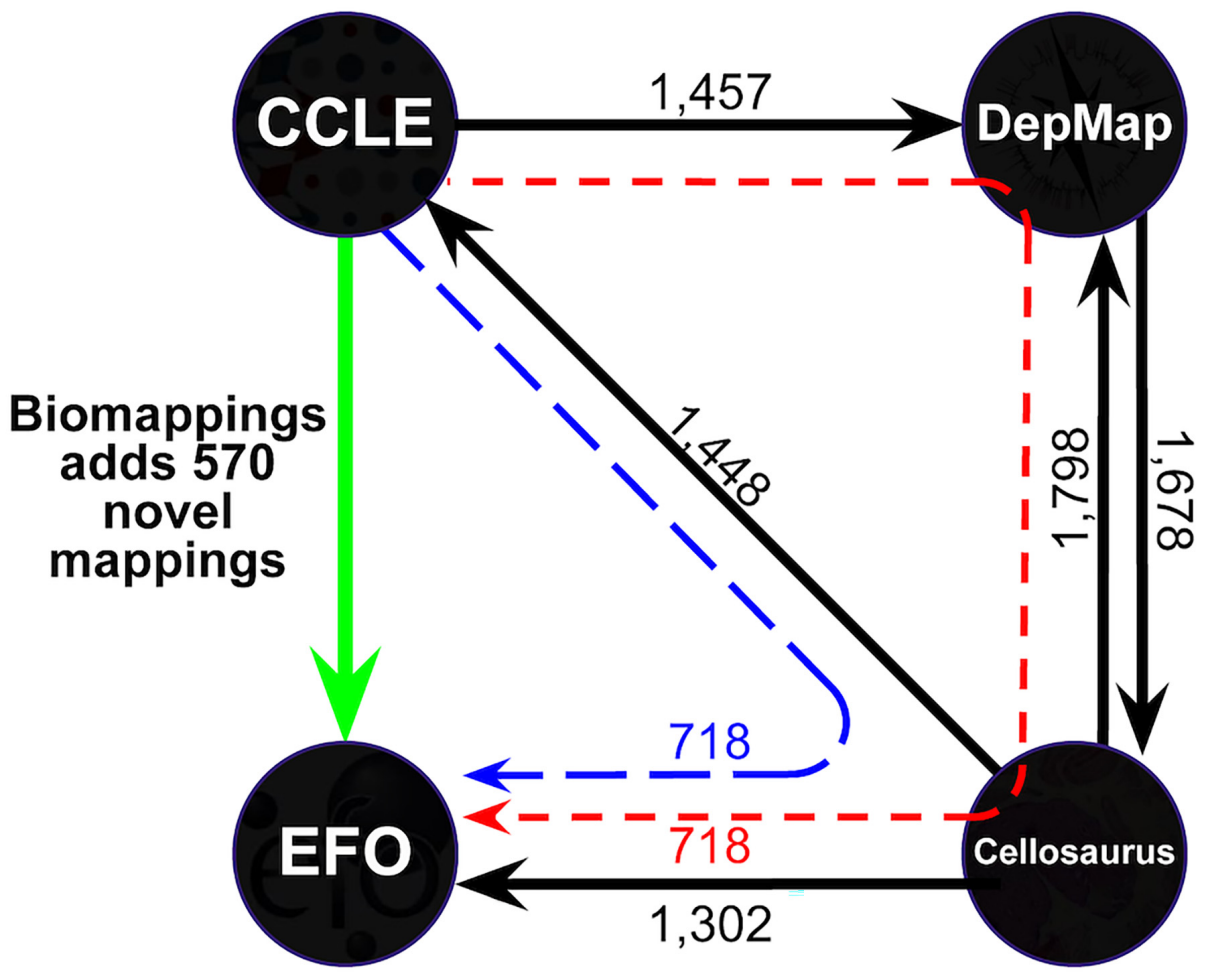
Mappings between identifiers in four cancer cell line resources. Solid black arrows represent primary mappings provided by identifier resources (arrows point from an identifier resource that provides a mappings to its target, labels show the number of mappings provided). Dashed arrows represent mappings inferred through the combination of primary mappings through two hops (blue, 718 mappings inferred) or three hops (red, 718 mappings inferred). The red and blue arrows represent partially overlapping sets of mappable entities. The green arrow represents the added benefit of including manually curated lexical mappings from Biomappings between CCLE and EFO, 1289 total with 570 not obtainable from existing primary mappings nor mappings inferred from primary mappings.

EFO (v3.49.0) contains no direct mappings to CCLE, but Cellosaurus (v43.0) contains 1448 (black arrows in Fig. 4). Cellosaurus also contains 1302 mappings to EFO which enables the inference of 718 two-hop mappings from CCLE to EFO (blue dashed line in Fig. 4). Further, we were able to recover a set of 718 three-hop mappings from CCLE to EFO when using the Cancer Dependency Map (DepMap) as an intermediate (red dashed line in Fig. 4, note that these 718 three-hop mappings are only partially overlapping with the 718 two-hop ones). However, such inference is inconvenient, relies on several implicit assumptions, and is still incomplete.

In order to complete the alignments between CCLE, EFO, and Cellosaurus, we first generated lexicalizations from CCLE names by exploiting the naming convention where the cell line name is postpended with the tissue of origin (e.g. CL14_LARGE_INTESTINE). From these names, we were able to extract the cell line name (e.g. CL14) with high precision for each record in CCLE. Then, we generated novel lexical mappings (i.e. that were not inferrable) and curated 114 positive mappings (+8%) from CCLE to Cellosaurus and 570 positive mappings (+79%) from CCLE to EFO. We also curated 59 predicted mappings that we found to be incorrect (i.e. the two terms cannot be considered equivalent) from CCLE to Cellosaurus and 10 incorrect predictions from CCLE to EFO. Explicitly storing these incorrect mappings as negative examples helps avoid future errors in prediction. For example, we flagged the proposed mapping of “CL14_LARGE_INTESTINE” (ccle:CL14_LARGE_INTESTINE, a colorectal adenocarcinoma) to “XPCS2BASV Cl-14” (cellosaurus:ZP40, a transformed fibroblast) as incorrect. We measured the overall precision of the lexical prediction pipeline for the 586 mappings predicted from CCLE to EFO identifiers which, based on our curation, yielded 570 correct, 10 incorrect, and 6 unsure mappings. This corresponds to a precision in the range 97%–98%, depending on the correctness of the six unsure mappings. We found that precision was lower for the 186 predicted CCLE to Cellosaurus mappings (between 63% and 68%) likely due to the fact that most possible mappings (1448) are already provided by Cellosaurus, and therefore the remaining space of possible novel predictions is small (and enriched for the most difficult cases), resulting in a larger proportion of spurious predictions. A more detailed analysis of these mappings is available at: https://github.com/biopragmatics/biomappings/blob/master/notebooks/curation-precision.ipynb.

While this case study focused on aligning cancer cell line terms, it represents an important first step in more generally improving the interoperability between cell line resources which support comparative analysis, generation and characterization of disease models, and many other efforts in drug discovery.

### 4.2 Chemical identifier mappings to improve clinical trials data integration

ClinicalTrials.gov is a database of 430 thousand clinical trials provided by the United States National Library of Medicine. These trials cover around 3600 unique interventions (e.g. small molecules) and 4200 unique conditions (e.g. diseases), both annotated in the database using MeSH terms. However, MeSH does not provide primary mappings to highly accessible identifier resources (e.g. ChEBI) that are necessary to support data integration with other popular datasets using different identifier resources. (Some mappings are available from MeSH to CAS and UNII, but these are not annotated well and these resources are relatively inconvenient to use.)

In this case study, we focused on generating and curating lexical mappings from MeSH to ChEBI. Many interventions appear across multiple clinical trials, so rather than exhaustively curating MeSH term mappings for all unmapped interventions, we prioritized curation by frequency of appearance across all clinical trials. Because the distribution of frequencies of interventions is long-tailed, we selected a subset of MeSH terms that represented 80% of the respective unmapped trial-intervention instances.

This resulted in the curation of mappings from 282 interventions’ MeSH terms to ChEBI that covered 80% (120 thousand) of the 150 thousand trial-intervention pairs with unmapped interventions. Note that some previous curations from MeSH to ChEBI already existed in Biomappings from a combination of undirected curation and various task-specific curations, causing this number to be lower than if we had conducted this curation at the beginning of the Biomappings project. Further, iterations of identifying the most valuable 80% of remaining unmapped interventions become successively less impactful due to the remaining interventions appearing in fewer trials.

To provide an unbiased estimate of the precision of the lexical mapping predictions between ChEBI and MeSH, we randomly selected 100 predicted mappings. Manual curation of these 100 mappings yielded 97 positive mappings, 2 negative mappings, and 1 unsure mapping. This corresponds to an estimated precision between 97% and 98%, depending on the correctness of the one unsure mapping. We provide additional details on this evaluation at: https://github.com/biopragmatics/biomappings/blob/master/notebooks/chemicals-unbiased-evaluation.ipynb.

Ultimately, Biomappings (v0.3.0) contains 2909 mappings from MeSH to ChEBI. This allows mapping interventions of 100 342 clinical trials (70.6% of all trials with at least one intervention MeSH annotation) to 995 unique chemicals. These mappings enable the previously difficult integration of clinical trials data from ClinicalTrials.gov with other resources using (or mappable to) ChEBI. For example, this enables the integration of ClinicalTrials.gov with chemical-protein bioactivities from ChEMBL (Gaulton et al. 2017) and protein-pathway memberships from WikiPathways in a knowledge graph that can be queried in a simple way to answer questions like “What are the protein targets modulated in a given clinical trial?” and “What are the pathways modulated in a given clinical trial?”.

### 4.3 Extending community ontologies with missing mappings

Several high-quality OBO ontologies cover similar topics/domains as MeSH and therefore maintain mappings to relevant MeSH terms. When reviewing mappings to MeSH in multiple widely used community ontologies, we found that mappings were incomplete. We therefore predicted (using an automated lexical approach) mappings from four OBO ontologies to MeSH outlined in Table 2.

**Table 2.** A summary of upstream contributions of 1378 MeSH mappings back to primary ontology resources.

Resource	Count	Link
DO	974	PR No. 1073
MONDO	190	PR No. 4930
UBERON (Haendel et al. 2014)	130	PR No. 2432
Cell Ontology (CL) (Diehl et al. 2016)	84	PR No. 1561

We then implemented automated scripts for inserting the new mappings into the respective version-controlled source files for each ontology in the OWL/XML, functional OWL, or OBO text formats, depending on the ontology. Finally, we made pull requests on the associated GitHub repository for each ontology to integrate the proposed mappings made using Biomappings through which ontology maintainers were able to add these contributions.

Contributing curated mappings upstream is important and highly impactful because it is propagated directly to users, generic services that consume ontologies such as Ubergraph (Balhoff et al. 2022), other services that consume mappings such as the Ontology Mapping Service (https://github.com/EBISPOT/OXO), and pipelines that build knowledge graphs [e.g. PheKnowLator (Callahan et al. 2020)]. Together, these form the basis for a large number of computational workflows used by researchers and engineers in academic, industrial, and research institutions.

## 5 Discussion

We presented Biomappings, a repository for community curated mappings between biomedical entities. Biomappings stores predictions and manual curations along with granular metadata and high-level semantics for each. It relies on an open data, open code, and open infrastructure philosophy combined with a governance strategy that fosters community contributions and engagement. It also explicitly encourages reuse and redistribution via its highly permissive CC0 license. Biomappings uses public infrastructure for quality assurance and distribution to promote transparency and increase trust.

### 5.1 Limitations

Biomappings enables the curation of missing mappings that are not available from primary identifier resources. In some cases, however, identifier resources have idiosyncratic curation guidelines for what constitutes a mapping, e.g. in how strictly two terms need to match to justify a mapping. This means that conflicts may arise if the curators of a primary identifier resource differ in their interpretation with what is curated via Biomappings. These can be resolved through community engagement and discussion, and—as demonstrated in this article—direct contribution to primary resources in a public space.

Further, even after completing an exhaustive curation campaign to create mappings between two identifier resources, it is still possible that new terms will be added, more complex operations will be performed such as the splitting or merging of terms, or changes will be made to the scope of a given term’s definition. The automation of predictions in Biomappings can facilitate keeping up with updates to identifier resources; however, automation does not yet extend to interpreting more complex changes to resources over time, so manual review is still required.

### 5.2 Extensibility

The initial focus of Biomappings is on entities in the biomedical domain. However, the tooling and philosophy can be readily adapted to domains outside of biomedicine, in fields where multiple overlapping identifier resources exist and need to be mapped for data integration. One such example is agriculture and agronomy: as a proof of concept, Biomappings includes 142 curations contributed to align concepts related to soil in the Agronomy Ontology (Arnaud et al. 2020) and Agronomy Vocabulary (Mietzsch et al. 2021). This demonstrates the possibility of the extension of Biomappings’ scope and the project’s ability to motivate external contribution.

In our case studies, we used a lexical approach to predict missing mappings between resources. However, the Biomappings curation workflow does not depend on a specific approach for generating potential mappings between identifiers in two resources. For instance, knowledge graph-based or machine learning-based methods such as those demonstrated by Berrendorf et al. (2020) can be readily used with Biomappings. Our future work will involve making use of such methods to find mappings not found via lexical alignment.

### 5.3 Future work on Biomappings

Following the initial development and curation of Biomappings, two ongoing challenges remain. First, there are multiple ways in which the (semi-)automated approach to the generation and curation of predictions could be improved. These include improving the data model to propagate information about the version of each identifier resource used for the generation of predictions, and enabling re-running generation workflows in an automated way, then notifying relevant curators when new content is available. In order to reduce curation burden, maximize return on curator time, and ultimately to scale curation of mappings, we will need to develop more accurate approaches for prediction, filtering, and prioritization.

The second challenge is to build, train, and engage a community of curators. One sub-challenge of this is to create curation interfaces that can be more easily deployed (or hosted) and seamlessly interact with git and GitHub (e.g. similar to the OntoDev suite, see https://github.com/ontodev) to support potential curators who might not be familiar with social software workflows or comfortable with version control. These steps may eventually enable more widespread crowd-sourced curation, which has recently been employed by Wong et al. (2021) and Ramsey et al. (2021). A second sub-challenge is to improve the ability to contribute content curated in Biomappings back to primary identifier resources. While we demonstrated this for four OBO ontologies, there are both technical challenges (e.g. the data are not curated in public version control) and social challenges (e.g. the maintainers are not receptive to contribution) for contributing content to primary resources. Distributing the burden of curation, e.g. using Biomappings, has substantial potential to improve ontologies which are used either directly or indirectly by most modern scientists.

### 5.4 Future vision

We envision the broader community of curators and developers who create and consume ontology mappings working in several directions to better support the tasks (e.g. ontology merging, entity alignment) that rely on mappings. First, we hope to see more resources adopting formats and minimum metadata standards like SSSOM to make their mappings more reusable (e.g. by assigning more precise predicates and including provenance information). Second, we hope to see these resources converging on external standards for the syntax and semantics used to communicate the entities and predicates appearing in mappings, such as the Bioregistry (Hoyt et al. 2022) in order to improve interoperability. Third, we hope to see large-scale efforts to aggregate, store, and redistribute mappings with more general scope than existing mapping services. Finally, we hope to see the development, implementation, and deployment of standardized, efficient algorithms for inference and retrieval of mappings, as well as an associated provenance model. We believe Biomappings will have an important role in supporting curation efforts and applications of mappings as the community works toward these goals.

## Supplementary Material

btad130_Supplementary_Data

## Data Availability

The data and code underlying this article are publicly available through GitHub at https://github.com/biopragmatics/biomappings under the MIT and CC0 licenses and are archived on Zenodo at https://doi.org/10.5281/zenodo.7307938.

## References

[btad130-B1] Allen J , de Beaumont W, Galescu L et al Complex event extraction using drum. In: Cohen B, Demner-Fushman D, Ananiadou S et al (eds), BioNLP 15, BioNLP Workshop Proceedings, pp. 1–11. Beijing: Association for Computational Linguistics, 2015.

[btad130-B2] Arnaud E , Laporte M-A, Kim S et al The ontologies community of practice: a CGIAR initiative for big data in agrifood systems. Patterns (N Y) 2020;1:100105. 33205138 10.1016/j.patter.2020.100105 PMC7660444

[btad130-B3] Bachman JA , Gyori BM, Sorger PK. Automated assembly of molecular mechanisms at scale from text mining and curated databases. Mol Syst Biol, 2023; e11325. 10.15252/msb.202211325 PMC10167483 36938926

[btad130-B4] Bairoch A. The Cellosaurus, a cell-line knowledge resource. J Biomol Tech 2018;29:25–38. 29805321 10.7171/jbt.18-2902-002 PMC5945021

[btad130-B5] Balhoff JP , Bayindir U, Caron AR et al Ubergraph: integrating OBO ontologies into a unified semantic graph. In: ICBO 2022, Ann Arbor, MI, USA, 2022.

[btad130-B6] Barretina J , Caponigro G, Stransky N et al The cancer cell line encyclopedia enables predictive modelling of anticancer drug sensitivity. Nature 2012;483:603–7. 22460905 10.1038/nature11003 PMC3320027

[btad130-B7] Berrendorf M , Faerman E, Melnychuk V et al Knowledge graph entity alignment with graph convolutional networks: lessons learned. In: ECIR 2020, Lisbon, Portugal, 12036 LNCS, pp. 3–11, Springer, 2020.

[btad130-B8] Bodenreider O , Zhang S. Comparing the representation of anatomy in the FMA and SNOMED CT. In: AMIA Annual Symposium Proceedings, Washington DC, USA, pp. 46–50, American Medical Informatics Association 2006. 17238300 PMC1839313

[btad130-B9] Callahan TJ , Tripodi IJ, Hunter LE et al A framework for automated construction of heterogeneous large-scale biomedical knowledge graphs. bioRxiv, 2020.

[btad130-B10] Diehl AD , Meehan TF, Bradford YM et al The cell ontology 2016: enhanced content, modularization, and ontology interoperability. J Biomed Semant 2016;7:44. 10.1186/s13326-016-0088-7 PMC4932724 27377652

[btad130-B11] Donnelly K et al SNOMED-CT: the advanced terminology and coding system for eHealth. Stud Health Technol Inform 2006;121:279. 17095826

[btad130-B12] Friedrichs M. Biodwh2: an automated graph-based data warehouse and mapping tool. J Integr Bioinform 2021;18:167–76. 33618440 10.1515/jib-2020-0033 PMC8238471

[btad130-B13] Gaulton A , Hersey A, Nowotka ML et al The ChEMBL database in 2017. Nucleic Acids Res 2017;45:D945–54. 27899562 10.1093/nar/gkw1074 PMC5210557

[btad130-B14] Geleta D , Nikolov A, ODonoghue M et al OntoMerger: an ontology integration library for deduplicating and connecting knowledge graph nodes. arXiv, 2022.

[btad130-B15] Ghandi M , Huang FW, Jané-Valbuena J et al Next-generation characterization of the cancer cell line encyclopedia. Nature 2019;569:503–8. 31068700 10.1038/s41586-019-1186-3 PMC6697103

[btad130-B16] Ghazvinian A , Noy NF, Musen MA. Creating mappings for ontologies in biomedicine: simple methods work. In: *AMIA … Annual Symposum proceedings, AMIA Symposium*, Washington DC, USA, pp. 198–202, American Medical Informatics Association 2009. PMC2815474 20351849

[btad130-B17] Guo X , Berrill A, Kulkarni A et al Merging ontologies algebraically. arXiv, 2022.

[btad130-B18] Gyori BM , Bachman JA, Subramanian K et al From word models to executable models of signaling networks using automated assembly. Mol Syst Biol 2017;13:954. 29175850 10.15252/msb.20177651 PMC5731347

[btad130-B19] Gyori BM , Hoyt CT, Steppi A. Gilda: biomedical entity text normalization with machine-learned disambiguation as a service. Bioinform Adv 2022;2:vbac034. 36699362 10.1093/bioadv/vbac034 PMC9710686

[btad130-B20] Haendel MA , Balhoff JP, Bastian FB et al Unification of multi-species vertebrate anatomy ontologies for comparative biology in uberon. J Biomed Semant 2014;5:21. 10.1186/2041-1480-5-21 PMC4089931 25009735

[btad130-B21] Hastings J , Owen G, Dekker A et al ChEBI in 2016: improved services and an expanding collection of metabolites. Nucleic Acids Res 2016;44:D1214–9. 26467479 10.1093/nar/gkv1031 PMC4702775

[btad130-B22] Hatos A , Quaglia F, Piovesan D et al APICURON: a database to credit and acknowledge the work of biocurators. Database 2021;2021:baab019. 10.1093/database/baab019 PMC8060004 33882120

[btad130-B23] Himmelstein DS , Lizee A, Hessler C et al Systematic integration of biomedical knowledge prioritizes drugs for repurposing. Elife 2017;6:e26726. 10.7554/eLife.26726 PMC5640425 28936969

[btad130-B24] Hoyt CT , Balk M, Callahan TJ et al Unifying the identification of biomedical entities with the bioregistry. Sci Data 2022;9:714. 36402838 10.1038/s41597-022-01807-3 PMC9675740

[btad130-B25] Ikeda S , Ono H, Ohta T et al TogoID: an exploratory ID converter to bridge biological datasets. Bioinformatics 2022;38:btac491. 10.1093/bioinformatics/btac491 PMC9438948 35801937

[btad130-B26] Jackson RC , Balhoff JP, Douglass E et al ROBOT: a tool for automating ontology workflows. BMC Bioinformatics 2019;20:407. 31357927 10.1186/s12859-019-3002-3 PMC6664714

[btad130-B27] Jackson RC , Matentzoglu N, Overton JA et al OBO foundry in 2021: operationalizing open data principles to evaluate ontologies. Database (Oxford) 2021;2021:1–9. 10.1093/database/baab069 PMC8546234 34697637

[btad130-B28] Jiménez-Ruiz E , Cuenca Grau B. LogMap: logic-Based and scalable ontology matching. In: Aroyo L, Welty C, Alani H et al (eds), The Semantic Web—ISWC 2011, pp. 273–88. Berlin, Heidelberg: Springer Berlin Heidelberg, 2011.

[btad130-B29] Kanehisa M , Furumichi M, Tanabe M et al KEGG: new perspectives on genomes, pathways, diseases and drugs. Nucleic Acids Res 2017;45:D353–61. 27899662 10.1093/nar/gkw1092 PMC5210567

[btad130-B30] Laadhar A , Abrahão É, Jonquet C. Investigating one million xrefs in thirty ontologies from the OBO world. In: Hastings J, Loebe F (eds), ICBO 2020, Vol. 2807 of CEUR Workshop Proceedings, Ann Arbor, MI, USA, pp. 1–12, 2020 (CEUR-WS.org).

[btad130-B31] Lambrix P , Tan H. 2008. Ontology Alignment and Merging. London: Springer, 133–49.

[btad130-B32] Maglott D , Ostell J, Pruitt KD et al Entrez Gene: gene-centered information at NCBI. Nucleic Acids Res 2011;39:52–7. 10.1093/nar/gkq1237 PMC3013746 21115458

[btad130-B33] Malone J , Holloway E, Adamusiak T et al Modeling sample variables with an experimental factor ontology. Bioinformatics 2010;26:1112–8. 20200009 10.1093/bioinformatics/btq099 PMC2853691

[btad130-B34] Martens M , Ammar A, Riutta A et al WikiPathways: connecting communities. Nucleic Acids Res 2021;49:D613–21. 33211851 10.1093/nar/gkaa1024 PMC7779061

[btad130-B35] Matentzoglu N , Balhoff JP, Bello SM et al A simple standard for sharing ontological mappings (SSSOM). Database2022a;2022:baac035. 35616100 10.1093/database/baac035 PMC9216545

[btad130-B36] Matentzoglu N , Goutte-Gattat D, Tan SZK et al Ontology development kit: a toolkit for building, maintaining and standardizing biomedical ontologies. Database2022b;2022:baac087. 36208225 10.1093/database/baac087 PMC9547537

[btad130-B37] Mietzsch E , Martini D, Kolshus K et al How agricultural digital innovation can benefit from semantics: the case of the AGROVOC multilingual thesaurus. Eng Proc 2021;9:17.

[btad130-B38] Nicholson DN , Greene CS. Constructing knowledge graphs and their biomedical applications. Comput Struct Biotechnol J 2020;18:1414–28. 32637040 10.1016/j.csbj.2020.05.017 PMC7327409

[btad130-B39] Pratt D , Chen J, Welker D et al NDEx, the network data exchange. Cell Syst 2015;1:302–5. 26594663 10.1016/j.cels.2015.10.001 PMC4649937

[btad130-B40] Ramsey J , McIntosh B, Renfro D et al Crowdsourcing biocuration: the community assessment of community annotation with ontologies (CACAO). PLoS Comput Biol 2021;17:e1009463. 34710081 10.1371/journal.pcbi.1009463 PMC8553046

[btad130-B41] Rogers FB. Medical subject headings. Bull Med Libr Assoc 1963;51:114–6. 13982385 PMC197951

[btad130-B42] Rosse C , Mejino JLV. A reference ontology for biomedical informatics: the foundational model of anatomy. J Biomed Inform 2003;36:478–500. 14759820 10.1016/j.jbi.2003.11.007

[btad130-B43] Schriml LM , Munro JB, Schor M et al The human disease ontology 2022 update. Nucleic Acids Res 2021;50:D1255–61. 10.1093/nar/gkab1063 PMC8728220 34755882

[btad130-B44] van Iersel MP , Pico AR, Kelder T et al The BridgeDb framework: standardized access to gene, protein and metabolite identifier mapping services. BMC Bioinformatics 2010;11:5. 20047655 10.1186/1471-2105-11-5 PMC2824678

[btad130-B45] Vasilevsky NA , Matentzoglu NA, Toro S et al Mondo: unifying diseases for the world, by the world. medRxiv, 2022.

[btad130-B46] Wang P , Hu Y. Matching biomedical ontologies via a hybrid graph attention network. Front Genet 2022;13:893409. 35938027 10.3389/fgene.2022.893409 PMC9354052

[btad130-B47] Wilding JL , Bodmer WF. Cancer cell lines for drug discovery and development. Cancer Res 2014;74:2377–84. 24717177 10.1158/0008-5472.CAN-13-2971

[btad130-B48] Wilkinson MD , Dumontier M, Aalbersberg IJ et al The FAIR guiding principles for scientific data management and stewardship. Sci Data 2016;3:160018. 26978244 10.1038/sdata.2016.18 PMC4792175

[btad130-B49] Wong JV , Franz M, Siper MC et al Science forum: author-sourced capture of pathway knowledge in computable form using biofactoid. Elife 2021;10:e68292. 34860157 10.7554/eLife.68292 PMC8683078

[btad130-B50] Yates B , Braschi B, Gray KA et al Genenames.org: the HGNC and VGNC resources in 2017. Nucleic Acids Res 2017;45:D619–25. 27799471 10.1093/nar/gkw1033 PMC5210531

[btad130-B51] Zerbino DR , Achuthan P, Akanni W et al Ensembl 2018. Nucleic Acids Res 2018;46:D754–61. 29155950 10.1093/nar/gkx1098 PMC5753206

